# Pasteurella multocida Bacteremia and Septic Arthritis Without an Animal Bite in an Older Patient Successfully Managed Without Joint Drainage: A Case Report

**DOI:** 10.7759/cureus.102807

**Published:** 2026-02-02

**Authors:** Masaatsu Kuwahara, Hideaki Imanaka

**Affiliations:** 1 Department of Emergency Medicine and Critical Care Medicine, Takarazuka City Hospital, Takarazuka, JPN

**Keywords:** antimicrobial therapy, bacteremia, cat exposure, conservative management, non-bite transmission, older patient, pasteurella multocida, septic arthritis

## Abstract

*Pasteurella multocida* is a Gram-negative coccobacillus commonly associated with animal bites or scratches. Invasive infections without a clear history of animal injury are uncommon, particularly in patients without overt immunosuppressive conditions. We report a case of *P. multocida* bacteremia and septic arthritis in an 82-year-old woman with domestic cat exposure but no history of a bite or scratch. The patient presented with fever and bilateral calf pain and was initially treated for sepsis of unknown origin. Blood and synovial fluid cultures subsequently identified *P. multocida*. She was successfully treated with antimicrobial therapy alone without joint drainage, and repeat synovial fluid cultures were negative. This case highlights that *P. multocida* should be considered as a potential pathogen in older patients with systemic infection and animal exposure, even in the absence of a bite injury, and that conservative management may be an option in carefully selected cases under close monitoring.

## Introduction

*Pasteurella multocida* is a small, Gram-negative, nonmotile coccobacillus that commonly colonizes the upper respiratory tract of domestic animals, particularly cats and dogs, as well as other mammals and birds [1]. In humans, infection most frequently occurs as localized soft tissue infection following animal bites or scratches, typically presenting as rapidly progressive cellulitis at the site of injury [2]. However, invasive infections such as bacteremia, meningitis, pneumonia, and septic arthritis have been reported, especially in patients with underlying conditions including liver cirrhosis, diabetes mellitus, malignancy, or other immunocompromised states [3].

Systemic *P. multocida* infection in patients without overt immunosuppressive conditions is uncommon, although it has been described in immunocompetent hosts with animal exposure [4]. We report a rare case of *P. multocida* bacteremia complicated by septic arthritis in an older patient with cat exposure but no history of an animal bite or scratch, who was successfully managed with antimicrobial therapy alone.

## Case presentation

Patient background

An 82-year-old woman with a medical history of mild dementia, epilepsy, and surgically treated rectal cancer presented with fever and bilateral calf pain. She had no known immunosuppressive conditions, had not received immunosuppressive medications, and had no history of alcohol consumption or smoking. She was taking lansoprazole 15 mg daily. The patient lived independently and owned a domestic cat, but denied any recent animal bites or scratches. She had no history of splenectomy.

Hospital course

Day 0

The patient was transported to the emergency department by ambulance. On admission, her body temperature was 38.3°C, blood pressure was 132/74 mmHg, pulse rate was 92 beats per minute, and oxygen saturation was 97% on room air. Her Glasgow Coma Scale score was 14 (E4V4M6). The Sequential Organ Failure Assessment (SOFA) score was 4, based on thrombocytopenia, renal dysfunction, and altered mental status.

Physical examination revealed bilateral calf tenderness without erythema, swelling, or skin discoloration. Laboratory testing showed a white blood cell count of 8,400/μL (reference range: 3,500-9,000/μL), an elevated C-reactive protein (CRP) level of 18.6 mg/dL (reference range: <0.3 mg/dL), and a serum creatinine level of 1.28 mg/dL (reference range: 0.46-0.79 mg/dL).

**Figure 1:**
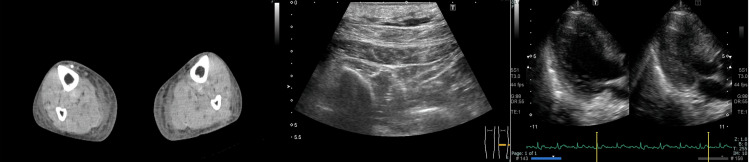
Imaging studies during hospitalization. Non-contrast computed tomography of the lower extremities (left) demonstrated diffuse soft tissue edema without gas formation or abscess, making necrotizing soft tissue infection unlikely. Lower limb ultrasonography (middle) revealed no evidence of deep vein thrombosis. Transthoracic echocardiography (right), including parasternal long-axis views of the left ventricle and mitral and aortic valves, showed no evidence of valvular vegetation or findings suggestive of infective endocarditis.

Non-contrast computed tomography of the lower extremities showed no evidence of gas formation or abscess, and other imaging studies revealed no deep vein thrombosis (DVT) or infective endocarditis (Figure 1).

Lower extremity venous ultrasonography revealed no evidence of DVT. No definite cause of the bilateral calf pain was identified. Empiric intravenous ceftriaxone (2 g once daily) was initiated for presumed sepsis of unknown origin.

Selected laboratory findings during hospitalization are summarized in Table 1.

**Table 1:** Selected laboratory findings during hospitalization WBC: white blood cell; CRP: C-reactive protein; PLT: platelet count; Hb: hemoglobin; Cr: creatinine; BUN: blood urea nitrogen; Na: sodium; K: potassium; Cl: chloride

Parameter	Reference Range	Day 0	Day 1	Day 3	Day 5	Day 7	Day 9	Day 11	Day 13	Day 15	Day 17	Day 19	Day 21
Cr (mg/dL)	0.46–0.79	1.71	1.38	0.96	0.83	0.8	0.74	0.73	0.73	0.75	0.76	0.75	0.78
Na (mmol/L)	138–145	145	149	150	153	147	144	136	135	138	141	140	141
K (mmol/L)	3.6–4.8	3.1	2.7	2.9	3	2.9	3.4	3.6	3.4	3.3	2.8	2.9	2.6
Cl (mmol/L)	101–108	108	113	109	111	107	103	97	98	102	102	102	102
CRP (mg/dL)	<0.3	27.22	27.83	17.06	14.53	12.86	11.55	13.65	8.9	11.83	11.19	7.16	6.43
WBC (×10³/µL)	3.5–9.0	11.34	9.88	9.41	9.61	7.79	7.05	6.6	8.04	6.78	5.79	4.82	5.21
PLT (×10⁴/µL)	15–35	93	92	101	142	151	160	204	202	214	209	203	202

Days 1-2

Blood cultures grew Gram-negative rods within 24 hours in both aerobic and anaerobic bottles. Urine culture suggested *Escherichia coli*; however, the blood culture isolate was considered a different organism. The patient remained febrile but hemodynamically stable, and ceftriaxone therapy was continued.

Day 3

The patient developed swelling, warmth, and pain in the left knee. Arthrocentesis revealed cloudy synovial fluid with numerous white blood cells and Gram-negative rods on Gram stain. Both blood and synovial fluid cultures identified *P. multocida*. β-lactamase production, which confers resistance to penicillin antibiotics, was not detected. Given the susceptibility profile, antimicrobial therapy was de-escalated to intravenous ampicillin (2 g every 8 hours). Transthoracic echocardiography showed no evidence of infective endocarditis.

Days 4-7

The patient’s fever gradually subsided, and her general condition improved; however, the decline in inflammatory markers was modest, with CRP decreasing from 18.6 mg/dL to 14 mg/dL. To broaden anaerobic coverage, as anaerobic coinfection cannot be completely excluded in septic arthritis even when cultures are negative, antimicrobial therapy was changed to intravenous ampicillin-sulbactam (3 g every 8 hours).

Days 10-14

The patient remained afebrile and clinically stable. CRP levels continued to decrease from 11.5 mg/dL to 8.9 mg/dL. Orthopedic consultation was obtained, and because the patient demonstrated continuous clinical improvement with antimicrobial therapy alone, conservative management without joint drainage was selected with close monitoring.

Day 16

Repeat synovial fluid culture was performed and confirmed to be negative.

Day 20

After further clinical and laboratory improvement, intravenous ampicillin-sulbactam was discontinued, and oral amoxicillin-clavulanate (500 mg/125 mg after each meal) was initiated for additional three weeks.

Day 22

The patient remained stable and afebrile and was discharged in good condition to a rehabilitation facility.

Microbiological findings

Blood cultures became positive within 24 hours in both aerobic and anaerobic bottles and yielded *P. multocida*. The organism was identified using standard automated microbiological methods, and β-lactamase production was not detected. Synovial fluid cultures also yielded *P. multocida*, with numerous white blood cells and evidence of crystal phagocytosis, suggesting concomitant calcium pyrophosphate deposition disease (CPPD). Antimicrobial susceptibility testing demonstrated susceptibility to all tested agents, including penicillins, β-lactam/β-lactamase inhibitor combinations, cephalosporins, carbapenems, and fluoroquinolones. Antimicrobial susceptibility testing results are shown in Table 2.

**Table 2:** Antimicrobial susceptibility of Pasteurella multocida MIC: minimum inhibitory concentration; S: susceptible ABPC: ampicillin; CCL: cefaclor; CPDX: cefpodoxime; CEZ: cefazolin; CTM: cefotiam; CTX: cefotaxime; CAZ: ceftazidime; CFPM: cefepime; CMZ: cefmetazole; IPM: imipenem; MEPM: meropenem; AZT: aztreonam; A/S: ampicillin-sulbactam; C/A: amoxicillin-clavulanate; P/T: piperacillin-tazobactam; GM: gentamicin; AMK: amikacin; MINO: minocycline; ST: trimethoprim-sulfamethoxazole; CPFX: ciprofloxacin; LVFX: levofloxacin; FOM: fosfomycin. β-lactamase production was not detected.

Antimicrobial Agent	MIC (µg/mL)	Interpretation
ABPC	≦2	S
CCL	≦4	S
CPDX	≦1	S
CEZ	1	S
CTM	≦2	S
CTX	≦0.25	S
CAZ	≦1	S
CFPM	≦2	S
CMZ	≦4	S
IPM	≦0.12	S
MEPM	≦0.12	S
AZT	≦1	S
A/S	≦2	S
C/A	≦2	S
P/T	≦8	S
GM	≦2	S
AMK	≦8	S
MINO	≦2	S
ST	≦20	S
CPFX	0.12	S
LVFX	≦0.12	S
FOM	≦32	S

## Discussion

*P. multocida* is most commonly associated with soft tissue infections following animal bites or scratches, but it can also cause invasive diseases, including bacteremia, endocarditis, respiratory infections, and bone and joint infections, particularly in infants and older adults [5]. Invasive infections without a clear history of animal injury are rare but have been reported, especially in older individuals, in whom comorbidities and age-related immune dysfunction are frequent [6]. Therefore, even in the absence of overt immunosuppressive conditions, advanced age itself should be regarded as a vulnerability factor rather than as strictly “immunocompetent” status.

Non-bite transmission of *P. multocida*, including infection following animal licking, respiratory droplets, or indirect contact with pet secretions, has been increasingly recognized. In a series of 79 pet-associated infections, 34 cases involved non-bite transmission, which occurred more often in older patients and were associated with more severe, life-threatening presentations than bite-associated infections [7]. Fatal non-bite bacteremia has also been reported in an elderly pet owner without recognized immunosuppression [6]. In the present case, the patient’s ownership of a domestic cat was considered the most likely source of infection despite the absence of a bite or scratch, which is consistent with these reports.

*P. multocida* is generally susceptible to penicillins, and penicillin or ampicillin is considered first-line therapy [8]. However, penicillin- and β-lactam-resistant, including β-lactamase-producing, human isolates have been documented. Consequently, β-lactam/β-lactamase inhibitor combinations such as ampicillin-sulbactam, amoxicillin-clavulanate, or piperacillin-tazobactam are recommended as empiric therapy in severe or invasive infections until susceptibility results are available [9]. In this case, antimicrobial therapy was appropriately de-escalated to ampicillin once susceptibility was confirmed. Because the initial decline in inflammatory markers was modest, escalation to ampicillin-sulbactam to broaden coverage was reasonable and in line with recommendations for invasive *P. multocida* disease.

Septic arthritis due to *P. multocida* has been reported after both bite and non-bite exposure and may progress to septic shock or death if not adequately treated [10]. Standard management consists of appropriate antimicrobial therapy combined with joint drainage or washout to provide source control [11]. Failure to improve with appropriate antibiotics alone should prompt suspicion of inadequate source control and early surgical intervention. In the present case, joint drainage was carefully considered. Orthopedic surgery was consulted promptly after the diagnosis of septic arthritis, and a multidisciplinary discussion was held. Because the patient exhibited continuous clinical improvement, decreasing inflammatory markers, and a stable general condition under antimicrobial therapy, conservative management without drainage was chosen under close observation. Joint washout was reserved as a contingency plan in case of clinical deterioration or lack of response to treatment. The patient ultimately achieved full recovery, and repeat synovial fluid cultures were negative.

While this favorable course resembles selected successfully managed cases, joint drainage should still be regarded as the standard of care, and conservative treatment should be reserved for carefully selected patients with clear and sustained improvement [12].

The presence of crystal phagocytosis suggested concomitant CPDD, which may have contributed to joint inflammation and potentially masked the diagnosis of septic arthritis. Septic arthritis due to *P. multocida* has been described in older patients initially thought to have degenerative or inflammatory joint disease, and delayed recognition can lead to severe outcomes, including fatal septic shock [9]. Therefore, septic arthritis should be considered even when crystal-induced arthritis is suspected, particularly in older individuals with animal exposure.

Limitations

This case report has several limitations. First, quantitative synovial fluid cell count analysis was not available, and the assessment of inflammatory cells was based on semi-quantitative Gram stain findings. Second, this is a single case report, and the favorable outcome achieved with conservative management without joint drainage cannot be generalized. Third, although repeat synovial fluid culture confirmed microbiological resolution, additional cytological or biochemical analyses at the time of repeat aspiration were not performed. Finally, imaging and microbiological findings were evaluated qualitatively rather than quantitatively, which may limit direct comparison.

## Conclusions

This case highlights that *P. multocida* can cause bacteremia and septic arthritis in older patients without overt immunosuppressive conditions or a history of animal bites. Clinicians should consider *P. multocida* in the differential diagnosis of Gram-negative bacteremia or septic arthritis in patients with animal exposure, even in the absence of a bite injury. Although joint drainage remains the standard treatment for septic arthritis, conservative management without drainage may be considered in carefully selected cases demonstrating a favorable clinical response under close monitoring.
